# A Method for LC-MS/MS Profiling of Coumarins in *Zanthoxylum zanthoxyloides* (Lam.) B. Zepernich and Timler Extracts and Essential Oils

**DOI:** 10.3390/molecules22010174

**Published:** 2017-01-22

**Authors:** Yoro Tine, Franck Renucci, Jean Costa, Alassane Wélé, Julien Paolini

**Affiliations:** 1 Université de Corse, UMR CNRS 6134 SPE, Laboratoire de Chimie des Produits Naturels, Campus Grimaldi, BP 52, F-20250 Corte, France; thomastine34@gmail.com (Y.T.); renucci@univ-corse.fr (F.R.); costa@univ-corse.fr (J.C.); 2 Laboratoire de Chimie Organique et Thérapeutique, Faculté de Médecine, Pharmacie et Odontologie, Université Cheikh Anta Diop, BP 5005 Dakar-Fann, Senegal; alassane.wele@ucad.edu.sn

**Keywords:** essential oil, coumarins, *Zanthoxylum zanthoxyloides*, LC-APCI-MS/MS

## Abstract

The metabolites from the coumarin class, present in tissues of plants belonging mainly to the Rutaceae and Apiaceae families, included compounds with high chemical diversity such as simple coumarins and furocoumarins. These health-promoting components are recognized for their valuable biological activities in herbal preparations but also for their phototoxic effects. In this work, a targeted liquid chromatography (LC) coupled with tandem mass spectrometry (MS^2^) was developed for the screening of 39 reference standards of coumarins and furocoumarins in essential oils and plant extracts. Chromatographic separation was accomplished on reversed phase column using water/acetonitrile as the mobile phase and detection was performed on a hybrid QqQ/linear ion trap spectrometer fitted with an atmospheric pressure chemical ionization (APCI) source operating in positive ion mode. This analytical approach was applied to investigate the coumarin compositions of fruit essential oils and methanolic extracts obtained from separated parts (fruit, leaf, stem, trunk, and root) of *Zanthoxylum zanthoxyloides*. Ten coumarins and six furanocoumarins were reported in this species and data analyses were used to assess the suitability of these compounds to the metabolomics-based differentiation of plant organs. The quantification criteria of the metabolites in extract samples included linearity, limit of quantification, limit of detection, and matrix effect were validated. As reported for other species of the Rutaceae family, the concentration of coumarins was drastically higher in *Z. zanthoxyloides* fruits than in other plant organs.

## 1. Introduction

Within plant metabolites, coumarins and furanocoumarins represent a wide group of structurally highly diverse compounds, present in tissues of plants belonging mainly to the Rutaceae [1,2,3,4], Apiaceae [4], and Fabaceae families [5]. This compound class is increasingly recognized as a valuable health-promoting constituent of edible plants and herbal plant preparations due to their wide spectrum of biological activities [6,7,8,9]. However, coumarins and furanocoumarins have also been linked to phototoxic [10], mutagenic [11], carcinogenic [12], and hepatotoxic [13] effects and inhibitory properties of cytochrome P450s [14]. Thus, the European Cosmetics Directive 76/768/EEC was recently modified, introducing for the first time a limit on the presence and use of the photosensitizing furocoumarins in cosmetics [15]. The Commission Directive 95/34/EC has also added a new restriction to annex II (entry N°358) as follows; “In sun protection and bronzing products, furocoumarines shall be below 1 mg/kg”. In a more recent proposal SCCNFP/0392/00 (Scientific Committee on Cosmetic and Non-Food-Products), this 1 mg/kg limit is proposed to be generalized to all cosmetic products [16]. In 2004, the European Food Safety Authority (EFSA) established a tolerable daily intake of coumarin of 0.1 mg/kg body weight based on its hepatotoxicity [17].

Plants of the *Zanthoxylum* genus, belonging to the Rutaceae family, are deciduous aromatic shrubs and trees, comprising about 200 species native to warm temperate and subtropical regions of the world [1]. From the *Zanthoxylum* species, over 76 coumarins have been isolated up to date. Xanthotoxin, a methoxylated furocoumarin, was first isolated in 1911 from an alcoholic extract of *Zanthoxylum zanthoxyloides* fruits [18] and their physicochemical properties were characterized by Thoms et al. (1911) [19]. Furthermore, two xanthotoxin and bergapten were reported in the fruits by Paris and Moyse-Mignon (1947) [20]. Thereafter, three simple coumarins (umbelliferone, 6,7-dimethylesculetin, and scopoletin) and five furocoumarins (imperatorin, xanthotoxin, bergapten, marmesin, and psoralen) were identified and quantified by gas chromatography coupled with mass spectrometry (GC-MS) in dichloromethane extracts of dried fruits [21]. In a more recent paper [22], the presence of coumarin-type compounds (6,7-dimethylesculetin, herniarin, psoralen, xanthotoxin, bergapten, isopimpinellin, and imperatorin) has been reported in the essential oils of *Z. zanthoxyloides* fruits. Other phytochemical studies on *Z. zanthoxyloides* revealed the presence of essential oils [22,23,24,25,26,27,28], alkaloids [29,30,31], flavonoids [32], and amides [21,29,33].

Although GC-MS is not the best choice for compounds that contain relatively polar or heat-labile substituents, one can make use of derivatization to get a better result [4]. Several high performance liquid chromatography (HPLC) based methods targeting commercial and isolated components were developed in order to monitor coumarins. Reversed phase HPLC coupled with UV detection (HPLC-UV) [3,4,34,35,36,37] or MS (LC-MS and LC-MS/MS) [2,3,4,34,35,37,38,39,40,41,42,43] are the most sensitive and selective methods for detecting coumarin derivatives. However, LC-UV does not allow the quantification of concentrations of less than 10 mg/L [36], such as those encountered in the analysis of complex mixtures, while LC-MS is used to quantify concentrations ranging from μg/L to 10 mg/L. One of the problems of working with LC-MS of natural products is the choice of the ionization technique. Thus, electrospray ionization (ESI) [2,34,35,37,38,39,40,41,42,43] or atmospheric pressure chemical ionization (APCI) [2,3,4,35,38] are the ionization sources commonly used for characterizing mass spectrometry coumarins. ESI is the technique of choice for polar and higher molecular weight compounds, while APCI is suitable for less polar compounds and those of lower molecular weight than ESI. Dugo et al. (2000) reported that coumarins and psoralens give a better response with APCI, while ions were not observed in ESI mode [38].

In the present study, an LC-MS^2^-based method was developed to dereplicate the possible presence of 39 commercially available coumarins and furocoumarins in *Z. zanthoxyloides* extracts. Following these results, this work was designed to validate a highly sensitive and selective method for the qualitative and quantitative determination of these components in the fruit essential oils and solvent extracts (fruits, leaves, and barks) of Senegalese *Z. zanthoxyloides* specie. LC-MS/MS data were also processed using Principal Component Analysis and Discriminant Analysis (PCA-DA) to assess the suitability of coumarin compounds to a metabolomics-based differentiation of plant organs. The application of this analytical approach led to the quality control of this commercial drug, traditionally used throughout Central and West Africa in abdominal and dental problems, sickle-cell disease, and skin disorders such as psoriasis and vitiligo [44,45].

## 2. Results and Discussion

### 2.1. Targeted LC-MS^2^ Method

**Figure 1:**
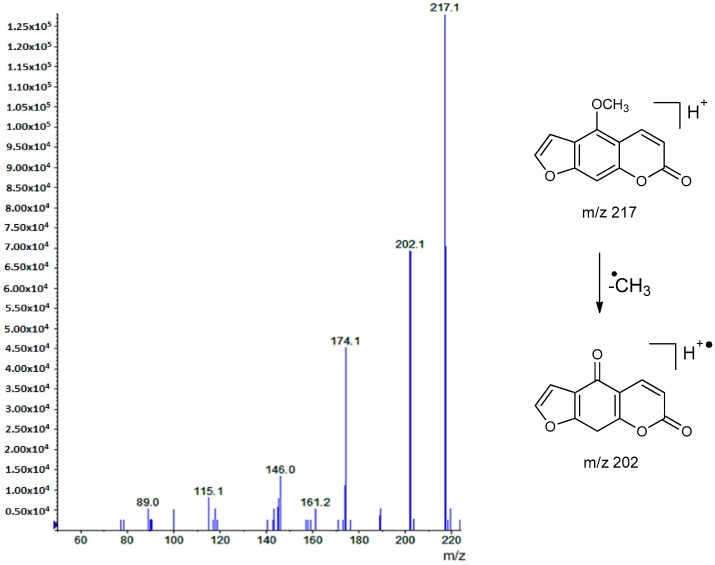
Enhanced product ion (EPI) mass spectrum of xanthotoxin with the atmospheric pressure chemical ionization (APCI) source in positive ion mode.

To obtain results on the structural diversity of coumarin-type components in plant extracts, an LC-MS^2^-based method was developed to detect the possible presence of 39 commercially available coumarins (31 standard compounds) and furocoumarins (8 standard compounds), which are characteristic of species from the Rutaceae family [1,2,3,4,14]. For each reference compound, a relevant transition of the precursor-to-product ions were detected with the utilization of the multiple reaction monitoring (MRM) mode. Using APCI source operating in positive mode, the precursor ion [M + H]^+^ for each of the 39 analytes was determined in MS1 full scan tests and the product ions in MS/MS experiments. MRM transitions of each analyte were optimized using direct infusion with the following MS/MS parameters; declustering potential (DP), entrance potential (EP), collision cell entrance potential (CEP), collision energy (CE), and collision cell exit potential (CXP) (Table 1). Retention times of reference compounds were determined by LC-MS^2^ analysis in the multiple reaction monitoring (MRM). Retention times of the 39 coumarin-type components are also indicated in Table 1. Mass spectra of standard components were performed by the MRM mode followed by an enhanced product ion (EPI) scan, triggered by information dependent acquisition (IDA) criteria. The fragmentation behavior of coumarins and furocoumarins was consistent with previous data reported in the literature [34,35,38,39,41,46]. Under collision-induced dissociation (CID), these compounds undergo neutral loss from pseudo-molecular ions [M + H]^+^, producing the corresponding high-abundance fragment ions: [M + H − CO]^+^, [M + H – CO − CO]^+^, [M + H − CO_2_]^+^, [M + H − C_5_H_9_O]^+^, [M + H − C_5_H_8_]^+^, [M + H − C_5_H_8_ − CO_2_]^+^, [M + H − CH_3_]^+^, and [M + H − 2CH_3_]^+^. For instance, the main ions (base peak) found on the mass spectra of the furocoumarin xanthotoxin was seen at *m*/*z* 217 [M + H]^+^ in Q1 and the predominant ion at *m*/*z* 202 ([M + H − CH_3_]^+^ (Figure 1).

### 2.2. Analysis of Z. zanthoxyloides Extracts Using LC-MS^2^ Method

**Figure 2:**
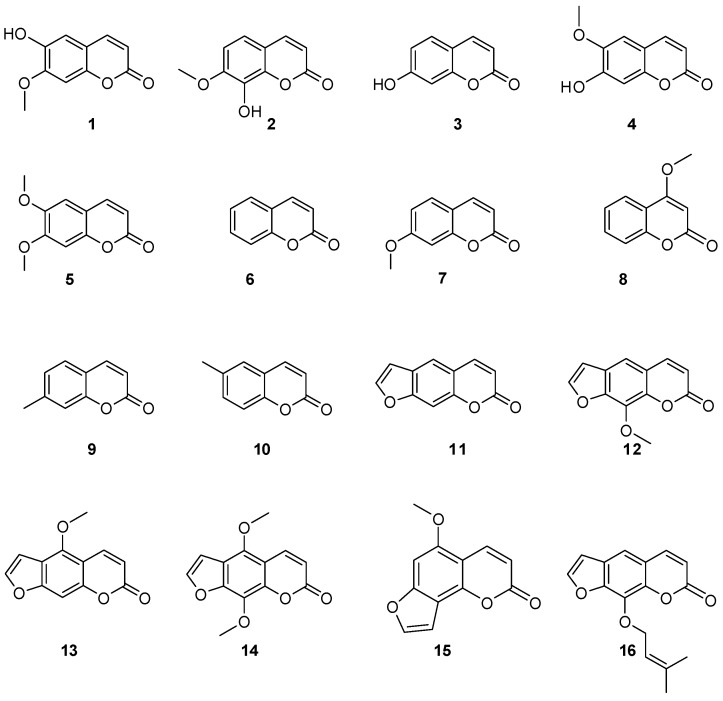
Chemical structures of coumarins (**1**–**10**) and furocoumarins (**11**–**16**) of *Z. zanthoxyloides* samples.

The identification of coumarins in *Z. zanthoxyloides* oils and extracts was allowed by the comparison of retention times, the observation of characteristic MRM transitions, and by matching the MS^2^ spectra of reference compounds (Table 1). The mobile phase, consisting of ACN/H_2_O (1:1 *v*/*v*), allowed the separation of targeted compounds of *Z. zanthoxyloides* extracts. Ten simple coumarins (**1**–**10**) and six furocoumarins (**11**–**16**) were unambiguously identified in all plant extracts (fruit, root bark, stem, and trunk bark) such as isoscopoletin **1**, daphnetin-7-methylether **2**, umbelliferone **3**, scopoletin **4**, 6,7-dimethylesculetin **5**, coumarin **6**, herniarin **7**, 4-methoxycoumarin **8**, 7-methylcoumarin **9**, 6-methylcoumarin **10**, psoralen **11**, xanthotoxin **12**, bergapten **13**, isopimpinellin **14**, isobergapten **15**, and imperatorin **16** (Figure 2). In addition to the detection of these sixteen components, no other standard compound was detected in the *Z. zanthoxyloides* oil and extracts.

**Figure 3:**
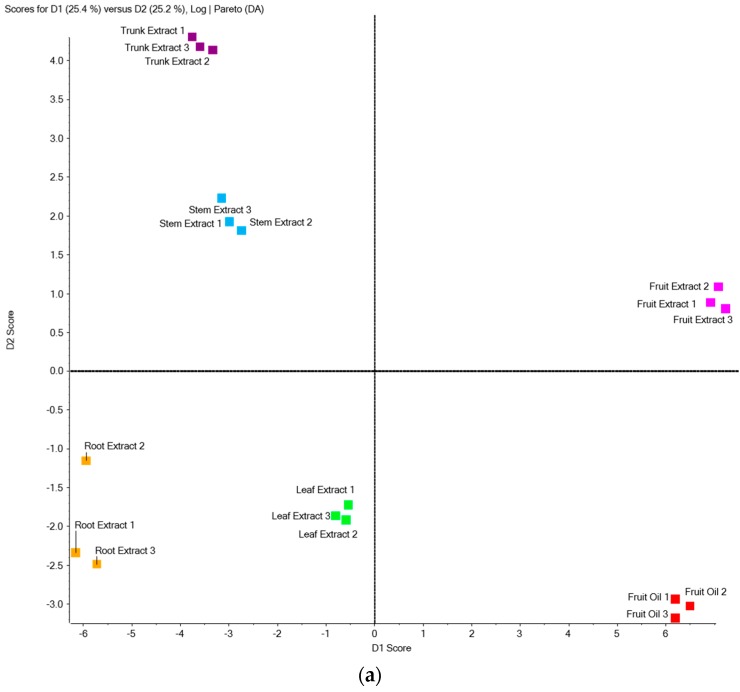

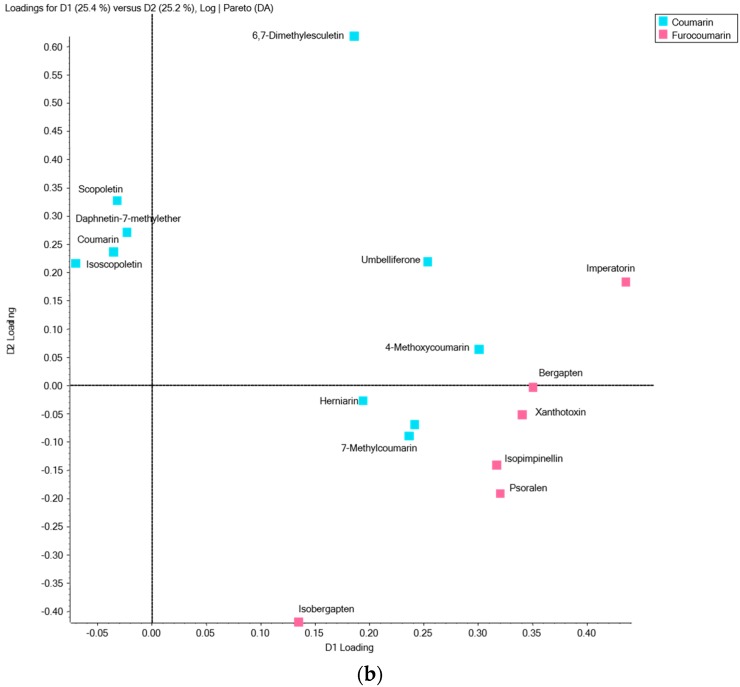
(**a**) Scores plot of principal component analysis and discriminant analysis (PCA-DA) of the *Z. zanthoxyloides* extracts (fruit, leaf, stem, root, trunk) and fruit essential oils analyzed using LC-MS/MS in the MRM mode; (**b**) Scores plot of PCA-DA of coumarins and furocoumarins detected in *Z. zanthoxyloides* samples using LC-MS/MS in the MRM mode.

In order to provide an evaluation of the variability of in *Z. zanthoxyloides* samples, essential oils and solvent extracts of each plant part were analyzed by LC-MS/MS, and MRM chromatograms were processed using PCA-DA. Targeted LC-MS profiles of the data set revealed separate clusters for essential oils and extracts, on the one hand, and differences associated with the plant parts, on the other hand. The PCA-DA analyses were based on calculations of intensity mean and standard deviation among the three replicates (methanolic extraction of each plant part collected on three distinct trees) of each peak, detected using scheduled MRM (Table S1). Using this statistical analysis, the scores plots of different *Z. zanthoxyloides* samples are displayed in Figure 3a. The principal factorial plane (constructed using D1 and D2) accounted for 50.6% of the entire variance of coumarins in plant samples. The fruit samples (essential oils and extracts) are correlated positively with the D1 axis (25.4%), whereas the extract samples of all other plant parts are negatively located on the D1 axis. Note that the trunk and stem samples are located in the same PCA space region, positively correlated with the D2 axis (25.2%), whereas the stem and root extract samples (located in the same quadrant) are negatively correlated with this axis. This indicates a potential differentiation of coumarin composition according to the *Z. zanthoxyloides* plant parts. The corresponding loading plot in Figure 3b shows the coumarins that make the most difference in separating samples. Characteristic marker components were identified to be responsible for clustering samples such as furocoumarins for fruit samples (essential oils and extracts) or scopoletin and isoscopoletin for trunk extracts.

From these results, it appeared that targeted LC-MS/MS, associated with statistical analysis using PCA-DA, is a rapid and effective approach to obtain similarities and/or differences in metabolic content among extraction methods and/or plant organs. In order to confirm PCA results and to obtain additional data for the differentiation of *Z. zanthoxyloides* plant parts according to coumarin-type metabolites, the quantification of these marker compounds was carried out by monitoring precursor-to-product ion transition MRM at specific retention times and by a comparison of peak areas in solvent extracts with those of calibration curves from corresponding standard compounds.

The calibration curves of most coumarins exhibited linearity in the concentration ranges (0.01–5 mg/L) with correlation coefficients higher than r^2^ ≥ 0.9934. The limit of quantification (LOQ) and limit of detection (LOD) of each compound were less than 0.1 mg/L and 0.03 mg/L. The detailed results of the regression equations, corresponding correlation coefficients, linear ranges, and LOQ are shown in Table 2. The matrix effect for 8-Acetyl-6-hydroxy-7-methoxycoumarin, used as the internal standard in plant extracts, ranged from −4.76% to +2.3% and fulfilled the criteria (≤15%).

The concentration of coumarins was much higher in *Z. zanthoxyloides* fruits as compared to other plant organs (Table 3). Indeed, five furocoumarins were found in high concentrations in the fruit extract, particularly xanthotoxin (**12**) and imperatorin (**16**) (39,522.3 mg/kg and 29,607.0 mg/kg respectively), followed by psoralen (**11**), bergapten (**13**), and isopimpinellin (**14**) (5192.6 mg/kg, 8786.8 mg/kg and 8439.3 mg/kg, respectively); this is in comparison with the main simple coumarins such as daphnetin-7-methyl ether (**2**), umbelliferone (**3**), and 6,7-dimethylesculetin (**5**) (1116.0 mg/kg, 1243.1 mg/kg and 1074.3 mg/kg, respectively). Seven other coumarin-type components (**1**, **4**, **7**–**10**, **15**) were reported in low amounts in the fruit extract. In comparison with the fruit extract, the contents of coumarins in other plant parts (leaves, roots, stems, and trunks) were very low (<3600 mg/kg) and umbelliferone (**3**), 7-methylcoumarin (**9**), and 6-methylcoumarin (**10**) were not found in these sample extracts. The presence of coumarin (**6**) was only reported in low amounts in the stem and trunk extracts. Finally, the coumarin composition of the trunk extracts exhibited relative high concentrations of simple coumarins, isoscopoletin (**1**), daphnetin-7-methylether (**2**), and scopoletin (**4**) in comparison to other plant organs. The amounts of coumarins and furocoumarins were drastically reduced by hydrodistillation, in comparison with methanolic extraction. For instance, the concentrations in the fruit essential oils of xanthotoxin (**12**) and imperatorin (**16**) (mg/kg dry plant) were 4.2 mg/kg and 2.8 mg/kg, respectively. The four main coumarin compounds in essential oils (mg/kg fruit oil) were xanthotoxin (**12**), imperatorin (**16**), psoralen (**11**), and bergapten (**13**); at 421.4 mg/kg, 284.4 mg/kg, 226.7 mg/kg, and 198.1 mg/kg, respectively.

These results are somewhat in agreement with previous studies conducted on fruit extracts of this species, which reported the presence of xanthotoxin, imperatorin, psoralen, bergapten, 6,7-dimethylesculetin, scopoletin, and umbelliferone using GC/MS [21]. Furthermore, the presence of two coumarins, xanthotoxin and bergapten, was detected by Paris and Moyse-Mignon in fruit extract (1947) [20]. It should be noted that all coumarin compounds identified in this study have been previously reported in the essential oils of other plants from the Rutaceae family, especially the *Citrus* species [2,3,35,38,40,43]. As indicated in the literature for other species of Rutaceae family such as *Ruta graveolens* [47], the concentration of these metabolites was drastically higher in *Z. zanthoxyloides* fruits than in other plant parts. To conclude, the use of the LC-MS/MS method, coupled with data analysis, is an effective analytical approach to obtain qualitative and quantitative results on the coumarin and furocoumarin compositions of plant extracts and essential oils. This study reported for the first time 9 coumarins (isoscopoletin, daphnetin-7-methylether, coumarin, herniarin, 4-methoxycoumarin, 7-methylcoumarin, 6-methylcoumarin, isopimpinellin, and isobergapten) in *Z. zanthoxyloides*. Finally, it appeared that the *Zanthoxylum* species, commonly used as health supplements in traditional medicine, are attractive for further investigations on coumarin-type compounds, particularly in terms of biological activity and the toxicity of plant extracts.

## 3. Materials and Methods

### 3.1. Solvents

Methanol (HPLC grade) and hexane (HPLC grade), used for sample extraction, were purchased from Fisher Scientific (Illkirch, France). The solvents used for liquid chromatography were LC-MS grade acetonitrile (ACN), obtained from Fisher Scientific. Deionized water was purified using a Milli-Q water (Millipore, Bedford, MA, USA) purification system.

### 3.2. Plant Material

The fruit, leaf, stem, and bark (root and trunk) samples of *Z. zanthoxyloides* were harvested in May 2015 (fruit ripening period) from three trees, growing wild in one Senegalese locality, Kafountine (12°56′5.49926″ N, 16°44′45.28315″ W). The botanical identification of the plant material was performed by Dr. William Diatta from the Department of botanical and pharmacognosy of University Cheikh Anta Diop of Dakar. A voucher specimen was deposited at the herbarium of that institution under number 001299.

### 3.3. Plant Extract and Essential Oil Preparation

Each plant organ (fruit, leaf, root bark, stem, and trunk bark) of each tree has been extracted separately. Plant samples were air dried for a period of four weeks at ambient temperature. The plant material was powdered with an average particle size of 0.2 mm using a blade miller (Polymix PX-MFC 90D, KINEMATICA AG, Luzern, Switzerland). 50 g of powder samples were extracted with 3 × 200 mL of methanol over 48 h each time, at room temperature under magnetic stirring. The solutions were combined, filtered through filter paper (PRATDUMAS, Couze-St-Front, France) and evaporated to dryness using a rotary evaporator (Laborota 4000, Heidolph, Schwabach, Germany). The methanolic solution was evaporated to dryness using a rotary evaporator and the extract yields (*w*/*w*, calculated on a dry weight plant) were 27.8%, 16.3%, 20.6%, 5.2%, and 14.2% for fruit, leaf, root bark, stem, and trunk bark, respectively. All dried extracts were stored at 4 °C until analysis. Fruit samples were also hydrodistillated (6 h) using a Clevenger-type apparatus (Midisciences, Fuveau, France) according to the method recommended in the European Pharmacopoeia [48]. The yield of essential oil (*w*/*w*, calculated on a dry weight basis) was 1.0% and the density was 0.86. Prior to LC-MS^2^ analysis, 10 mg of each sample extract was dissolved in ACN/H_2_O (1:1 *v*/*v*) to obtain a solution at a final concentration of 100 mg/L. The fruit oil was diluted to 1/100 in acetonitrile. Finally, the solutions were filtered on a 0.2 μm polytetrafluoroethylene (PTFE) filter (Whatman, Maidstone, UK). 1.4 mL of leaf, root, stem, and trunk extract solutions and essential oil solutions were supplemented with 0.1 mL of 8-Acetyl-6-hydroxy-7-methoxycoumarin, used as the internal standard at a concentration of 20 mg/L. For fruit extract, 0.3 mL of the solution was supplemented with 1.1 mL ACN/H_2_O (1:1 *v*/*v*) and 0.1 mL of the internal standard.

### 3.4. References Compounds and Preparation of Standard Solutions

All references of coumarins 4-methyldaphnetin, esculetin, 6-hydroxycoumarin, isoscopoletin, 6,7-dihydroxy-4-methylcoumarin, daphnetin-7-methylether, umbelliferone, scopoletin, 5,7-dihydroxy-4-methylcoumarin, 8-acetyl-6-hydroxy-7-methoxycoumarin, fraxidin, xanthotoxol, 6,7-dimethylesculetin, coumarin, 8-acetyl-7-methoxycoumarin, herniarin, 4-methoxycoumarin, 8-acetyl-6,7-dimethoxycoumarin, 3-acetylcoumarin, 7-methylcoumarin, psoralen, nordalbergin, 6-methoxy-4-methylcoumarin, 7-methoxy-4-methylcoumarin, xanthotoxin, 6-methylcoumarin, dalbergin, citropten, bergapten, isopimpinllin, 7-ethoxycoumarin, 4-hydroxycoumarin, 4-ethoxycoumarin, 4-methylumbelliferone, 4-methyl-7-ethoxycoumarin, isobergapten, bergaptol, imperatorin, and osthol (98% purity determined by HPLC) were purchased from Extrasynthese (Geney, France). Solutions of each standard were prepared by dissolving the reference compound in ACN/H_2_O (1:1 *v*/*v*) at a final concentration of 5 mg/L, which was then filtered on a 0.2 μm PTFE filter. These standard solutions were diluted with ACN/H_2_O (1:1 *v*/*v*) to obtain calibration curves with seven points in the concentration range of 0.01–5 mg/L. The calibration solutions were stored at 4 °C until LC-MS^2^ analysis. A blending solution, which contained the 39 reference components at a concentration of 0.1 mg/L in ACN/H_2_O (1:1 *v*/*v*), was used as positive control of LC-MS^2^ analysis of the plant extracts (before and after sample injections).

### 3.5. MS^2^ Conditions

MS^2^ conditions were carried on an AB Sciex (Toronto, ON, Canada) 3200 QTRAP linear triple quadrupole fitted with an atmospheric pressure chemical ionization (APCI) ion source operating in positive mode. High purity nitrogen was used as both a nebulizer and turbo gas. The APCI source was operated with following settings in positive mode; curtain gas (CUR) 25 psi, nebulizer gas (GS1) 31 psi, heater gas (GS2) 65 psi, ion spray voltage (IS) 5000 V, and temperature 450 °C. Standard solutions (component concentration: 0.1 mg/L) were directly infused at the flow rate of 10 μL/min in the MS/MS apparatus. Multiple EPI mass spectra of each compound were recorded in the range of *m*/*z* = 50–500 at 4000 Da/s. IDA properties were set to select 1 to 2 peaks above 300 counts with an exclusion filter after 5 occurrences for 30 s with dynamic background subtraction. The software used for data acquisition and data analysis was Analyst 1.5.2 (AB Sciex, Framingham, MA, USA).

### 3.6. LC Conditions

The LC system consisted of a Flexar LC Perkin-Elmer (Waltham, MA, USA) with two Flexar FX-10 LC pumps, a Flexar solvent manager, a 275-Flexar autosampler, and a Flexar LC PE200 column oven. LC analyses were performed on a 100 mm × 2.1 mm i.d. 3 μm, LUNA 3U C18 column (Phenomenex, Torrance, CA, USA) and the column temperature was set at 25 °C. A volume of 10 μL of sample was injected using an injection loop of 15 μL in partial loop mode. The mobile phase consisted of MilliQ water (solvent A) and ACN (solvent B). The flow rate was set at 500 μL/min. The column was equilibrated (A:B; *v*/*v*) in 90:10 (5 min), and elution was carried out with the following steps; 90:10 (5 min), 80:20 (5 min), 70:30 (5 min), 60:40 (5 min), 50:50 (5 min), a linear gradient increasing from 50% B to 100% (5 min), and 100% B (7 min).

### 3.7. LC-MS^2^ Quantification

The LC-MS^2^ method was validated according to the US Food and Drug Administration (FDA) guidance for bioanalytical method validation by assessing linearity, limit of quantification (LOQ), limit of detection (LOD), and matrix effect [49]. External standard calibration lines were generated by three repeated injections of standard solutions at seven concentration levels (0.01; 0.1; 0.25; 0.5; 1; 2.5; and 5 mg/L) at 1 day. A plot of peak area with respect to the corresponding concentration was used to demonstrate linearity. The linear regression equation and correlation coefficient were calculated by weighted (1/*x*^2^) least-squares linear regression analysis. Linearity was considered to be acceptable when correlation coefficients were 0.99 or better and calibrators had accuracies of 85%–115% and precisions within ± 15% RSD (relative standard deviation). The LOD (signal-to-noise <3) was defined as the amount that could be detected, while the LOQ (signal-to-noise >10) was the lowest concentration point of calibration curve at which accuracy (relative error, RE) within 20% and precision below 20% can be considered acceptable. The absolute matrix effect was evaluated by comparing the chromatographic peak areas of 8-Acetyl-6-hydroxy-7-methoxycoumarin (internal standard) in real sample extracts with those of 8-Acetyl-6-hydroxy-7-methoxycoumarin present in the “neat” mobile phase. The matrix effect is considered to be obvious if the ratio is less than ±15% [50].

### 3.8. Statistical Analysis

The triplicates of each methanol extract (fruit, leaf, stem, root, and trunk barks) and the essential oil from the fruit were analyzed by LC-MS/MS in the multiple reaction monitoring (MRM). MRM-MS chromatographic profiles of each sample were performed using PCA and DA in Marked View™ software (Sciex, Toronto, ON, Canada). PCA (principal components analysis) performs plans (principal components, PCs) where both objects (plant samples) and variables (coumarins components) are plotted according to the variance present in the data set. Discriminant analysis (DA) is performed in order to sharpen the separation between groups of observations, by hopefully rotating PCA components such that a maximum separation among plant samples is obtained, and to understand which coumarin variables carry the sample separating information.

## Figures and Tables

**Table 1:** Retention times (Tr), multiple reaction monitoring (MRM) transition, and optimized tandem mass spectrometry (MS/MS) detection parameters of 39 coumarins.

Compounds	Tr (min)	Transition		MS Parameters (V)				
		Q1 Mass (Da)	Q3 Mass (Da)	DP	EP	CEP	CE	CXP
4-Methyldaphnetin	9.5	193.1	119.2	56	8.0	12	31	4
Esculetin	11.7	179.0	123.1	71	9.0	10	27	4
6-Hydroxycoumarin	13.6	163.0	107.2	51	5.5	16	31	4
Isoscopoletin	14.1	193.1	133.1	56	12.0	12	25	4
6,7-Dihydroxy-4-methylcoumarin	14.3	193.1	91.2	76	10.5	10	37	4
Daphnetin 7-methylether	14.4	193.1	178.1	66	10.0	12	27	4
Umbelliferone	14.5	163.0	107.2	56	9.5	12	29	4
Scopoletin	14.5	193.1	133.2	76	8.5	10	25	4
5,7-Dihydroxy-4-methylcoumarin	14.6	193.1	91.1	66	11.0	10	39	4
8-Acetyl-6-hydroxy-7-methoxycoumarin	15.6	235.1	189.1	56	7.5	14	17	4
Fraxidin	15.7	223.1	190.1	61	9.5	14	19	4
Xanthotoxol	17.9	203.1	147.1	76	10.5	12	27	4
6,7-Dimethylesculetin	17.9	207.1	151.2	61	4.5	12	29	4
Coumarin	18.9	147.0	91.1	46	4.5	12	29	4
8-Acetyl-7-methoxycoumarin	18.9	219.1	115.2	61	8.5	12	43	4
Herniarin	20.8	177.1	121.1	56	4.0	12	27	4
4-Methoxycoumarin	20.9	177.1	118.1	61	3.0	12	27	4
8-Acetyl-6,7-dimethoxycoumarin	21.4	249.1	115.2	56	10.5	14	43	4
3-Acetylcoumarin	21.6	189.1	115.1	41	8.5	12	37	4
7-Methylcoumarin	22.7	161.1	105.1	51	8.5	12	29	4
Psoralen	22.7	187.1	131.1	56	10.5	12	33	4
Nordalbergin	22.7	255.1	152.2	76	10.0	14	55	4
6-Methoxy-4-methylcoumarin	22.8	191.1	91.2	71	5.0	12	51	4
7-Methoxy-4-methylcoumarin	23.2	191.1	91.2	71	4.0	12	53	4
Xanthotoxin	23.2	217.1	202.0	71	12.0	14	61	4
6-Methylcoumarin	23.7	161.0	105.1	46	9.0	10	27	4
Dalbergin	24.2	269.1	152.2	91	10.5	16	59	4
Citropten	24.6	207.1	121.3	61	10.5	12	33	4
Bergapten	24.7	217.1	202.0	61	8.5	14	27	4
Isopimpinllin	24.9	247.1	217.1	71	10.5	14	23	4
7-Ethoxycoumarin	25.0	191.1	163.1	56	4.5	12	25	4
4-Hydroxycoumarin	25.1	163.0	121.1	81	9.0	10	25	4
4-Ethoxycoumarin	25.2	191.1	163.0	46	9.0	12	21	4
4-Methylumbelliferone	26.3	177.1	77.1	91	12.0	12	45	4
4-Methyl-7-ethoxycoumarin	26.8	205.1	177.1	61	8.5	12	19	4
Isobergapten	28.7	217.1	202.1	61	8.5	12	33	4
Bergaptol	29.6	203.1	147.2	66	4.5	12	29	4
Imperatorin	31.5	271.2	203.1	51	5.0	14	17	4
Osthol	32.3	245.1	189.1	56	4.5	14	17	4

DP = declustering potential, EP = entrance potential, CEP = collision cell entrance potential, CE = collison energy, CXP = collision cell exit potential.

**Table 2:** Regression equations, correlation coefficients, and linear ranges of 16 coumarins (**1**–**16**) identified in the *Z. zanthoxyloides* samples.

No.	Compounds	Regression Equation	r^2^	Linear Range (mg/L)	LOQ (mg/L)	LOD (mg/L)
**1**	Isoscopoletin	*y* = 112,000*x* + 3270	0.9970	0.10–2.5	0.10	0.03
**2**	Daphnetin-7-methylether	*y* = 27,200*x* + 1260	0.9934	0.10–1.0	0.10	0.03
**3**	Umbelliferone	*y* = 705,000*x* + 1580	0.9991	0.01–5.0	0.01	0.003
**4**	Scopoletin	*y* = 119,000*x* + 2450	0.9961	0.10–5.0	0.10	0.03
**5**	6,7-Dimethylesculetin	*y* = 2,090,000*x* − 21,000	0.9974	0.10–5.0	0.10	0.03
**6**	Coumarin	*y* = 241,000*x* − 2300	0.9979	0.01–5.0	0.01	0.003
**7**	Herniarin	*y* = 4,080,000*x* − 3140	0.9989	0.01–5.0	0.01	0.003
**8**	4-Methoxycoumarin	*y* = 1,830,000*x* − 18,300	0.9941	0.01–5.0	0.01	0.003
**9**	7-Methylcoumarin	*y* = 1,170,000*x* − 2100	0.9973	0.10–5.0	0.10	0.03
**10**	6-Methylcoumarin	*y* = 874,000*x* − 11,500	0.9996	0.01–5.0	0.01	0.003
**11**	Psoralen	*y* = 2,150,000*x* − 3000	0.9981	0.01–5.0	0.01	0.003
**12**	Xanthotoxin	*y* = 1,180,000*x* + 472	0.9997	0.01–5.0	0.01	0.003
**13**	Bergapten	*y* = 4,330,000*x* − 1850	0.9989	0.01–1.0	0.01	0.003
**14**	Isopimpinellin	*y* = 3,330,000*x* + 721	0.9985	0.01–5.0	0.01	0.003
**15**	Isobergapten	*y* = 2,690,000*x* − 1410	0.9976	0.01–5.0	0.01	0.003
**16**	Imperatorin	*y* = 1,04,000*x* − 89	0.9951	0.01–5.0	0.01	0.003
**IS**	8-Acetyl-6-hydroxy-7-methoxycoumarin	*y* = 117,000*x* − 23.7	0.9992	0.01–2.5	0.01	0.003

In the regression equation *y* = a*x* + b, *x* refers to the sample injection amount, *y* to the peak area; r^2^ is the correlation coefficient of the equation, and LOQ is the limit of quantification.

**Table 3:** Contents of the coumarins and furocoumarins in *Z. zanthoxyloides* plant parts (*n* = 3).

No.	Components	Concentration of Coumarin Components (mg/kg Dry Plant Material Weight ± SD)						Concentration of Coumarin Components (mg/kg Fruit Oil Weight ± SD)
		Solvent Extracts					Essential Oil	
		Fruit	Leaf	Root	Stem	Trunk	Fruit	
**1**	Isoscopoletin	632.5 ± 20.3	<LOQ	<LOQ	118.3 ± 3.8	1047.8 ± 18.4	ND	ND
**2**	Daphnetin-7-methylether	1116.0 ± 21.6	ND	<LOQ	99.4 ± 6.5	1835.9 ± 35.1	<LOQ	<LOQ
**3**	Umbelliferone	1243.1 ± 26.9	ND	ND	ND	ND	ND	ND
**4**	Scopoletin	370.6 ± 8.6	<LOQ	<LOQ	<LOQ	577.8 ± 3.1	ND	ND
**5**	6,7-Dimethylesculetin	1074.3 ± 4.6	<LOQ	<LOQ	<LOQ	1062.0 ± 5.3	<LOQ	<LOQ
**6**	Coumarin	ND	ND	ND	<LOQ	<LOQ	0.1 ± 0.0	8.1 ± 0.6
**7**	Herniarin	152.0 ± 2.2	ND	ND	ND	79.3 ± 1.4	0.1 ± 0.0	6.5 ± 0.0
**8**	4-Methoxycoumarin	<LOQ	ND	ND	ND	<LOQ	tr	2.0 ± 0.0
**9**	7-Methylcoumarin	<LOQ	ND	ND	ND	ND	tr	3.4 ± 0.1
**10**	6-Methylcoumarin	<LOQ	ND	ND	ND	ND	tr	4.4 ± 0.1
**11**	Psoralen	5192.6 ± 68.8	59.1 ± 2.3	ND	<LOQ	ND	2.3 ± 0.1	226.7 ± 6.2
**12**	Xanthotoxin	39,522.3 ± 9.3	263.7 ± 9.1	<LOQ	13.5 ± 0.5	<LOQ	4.2 ± 0.1	421.4 ± 12.5
**13**	Bergapten	8786.8 ± 29.8	84.7 ± 0.5	ND	6.9 ± 0.1	ND	3.00 ± 0.0	198.1 ± 2.0
**14**	Isopimpinellin	8439.3 ± 13.8	35.1 ± 1.4	<LOQ	<LOQ	<LOQ	0.4 ± 0.0	39.2 ± 0.0
**15**	Isobergapten	99.9 ± 1.4	<LOQ	ND	ND	ND	tr	1.5 ± 0.1
**16**	Imperatorin	29,607.0 ± 0.0	224.1 ± 9.9	<LOQ	16.5 ± 0.1	<LOQ	2.8 ± 0.1	284.4 ± 6.5

ND: Not Dected; LOQ: Limit of Quantification; SD: Standard Deviation; tr: trace <0.1 mg/kg.

## Supplementary Materials

Supplementary materials can be accessed at: http://www.mdpi.com/1420-3049/22/1/174/s1. Table S1 Targeted LC-MS/MS profiles of *Z. zanthoxyloides* extracts.
