# Supplementary material for: A Method for LC-MS/MS Profiling of Coumarins in Zanthoxylum zanthoxyloides (Lam.) B. Zepernich and Timler Extracts and Essential Oils
Source: Molecules. 2017 Jan 22;22(1):174. doi: 10.3390/molecules22010174 (PMC6155696; doi:10.3390/molecules22010174)
Supplement: Supplementary file 1 [file molecules-22-00174-s001.pdf]

Supplementary Materials: A Method for LC-MS/MS Profiling of Coumarins in *Zanthoxylum zanthoxyloides* (Lam.) B. Zepernich and Timler Extracts and Essential Oils

Yoro Tine, Franck Renucci, Jean Costa, Alassane Wélé and Julien Paolini

Table S1. Targeted LC-MS/MS profiles of *Z. zanthoxyloides* extracts obtained among the three replicates (methanolic extraction of each plant part collected on three distinct trees). Intensity of scheduled MRM transition of each coumarin compound were used for PCA-DA analyses

Sample Name	Coumarin	6-Methylcoumarin	7-Methylcoumarin	Umbelliferone	Herniarin	4-Methoxycoumarin	Psoralen	Daphnetin-7-methylether	Isoscopoletin	6,7-Dimethylesculetin	Xanthotoxin	Bergapten	Isobergapten	Isopimpinellin	Imperatorin	Scopoletin
Fruit extract 1	2.23×10^3	5.28×10^3	5.23×10^3	4.41×10^4	2.16×10^4	2.31×10^3	5.77×10^5	1.00×10^3	2.66×10^3	8.30×10^4	1.52×10^6	1.22×10^6	2.20×10^3	9.92×10^5	1.65×10^5	1.44×10^3
Fruit extract 2	1.64×10^3	4.55×10^3	4.82×10^3	4.18×10^4	2.22×10^4	2.55×10^3	5.04×10^5	9.97×10^2	2.65×10^3	7.55×10^4	1.51×10^6	1.29×10^6	2.10×10^3	9.58×10^5	1.54×10^5	1.31×10^3
Fruit extract 3	1.36×10^3	4.91×10^3	5.04×10^3	4.21×10^4	2.00×10^4	2.41×10^3	4.68×10^5	1.02×10^3	2.93×10^3	7.26×10^4	1.45×10^6	1.29×10^6	2.12×10^3	9.64×10^5	1.69×10^5	1.34×10^3
Leaf extract 1	4.19×10^2	3.35×10^2	2.22×10^2	3.04×10^2	9.73×10^2	9.94×10^1	1.03×10^4	5.33×10^1	1.16×10^2	8.62×10^2	2.44×10^4	2.23×10^4	1.22×10^2	7.51×10^3	1.85×10^3	6.93×10^1
Leaf extract 2	3.59×10^2	3.02×10^2	2.08×10^2	3.04×10^2	8.90×10^2	1.14×10^2	9.76×10^3	4.56×10^1	1.23×10^2	1.05×10^3	2.54×10^4	2.20×10^4	8.56×10^1	7.27×10^3	1.73×10^3	6.93×10^1
Leaf extract 3	3.00×10^2	2.21×10^2	1.87×10^2	3.99×10^2	8.98×10^2	1.10×10^2	1.01×10^4	5.33×10^1	9.14×10^1	9.29×10^2	2.59×10^4	2.29×10^4	7.24×10^1	7.28×10^3	1.64×10^3	7.04×10^1
Root extract 1	1.94×10^2	1.58×10^2	1.65×10^2	1.18×10^2	4.26×10^2	5.59×10^1	5.49×10^2	9.50×10^1	2.75×10^2	2.27×10^2	7.04×10^2	6.50×10^2	9.00×10^1	4.26×10^2	7.51×10^1	1.10×10^2
Root extract 2	2.09×10^2	1.70×10^2	1.76×10^2	1.37×10^2	3.89×10^2	5.33×10^1	4.25×10^2	1.38×10^2	2.40×10^2	2.01×10^2	7.54×10^2	6.86×10^2	6.46×10^1	3.94×10^2	6.74×10^1	9.34×10^1
Root extract 3	2.00×10^2	1.58×10^2	1.60×10^2	1.33×10^2	4.79×10^2	4.68×10^1	4.87×10^2	1.45×10^2	2.66×10^2	2.14×10^2	7.64×10^2	6.69×10^2	6.49×10^1	4.94×10^2	6.74×10^1	1.33×10^2
Stem extract 1	1.86×10^2	1.97×10^2	2.08×10^2	2.28×10^2	5.23×10^2	8.38×10^1	8.92×10^2	3.46×10^2	8.67×10^2	1.59×10^4	4.62×10^3	5.81×10^3	3.88×10^1	1.62×10^3	3.49×10^2	4.93×10^2
Stem extract 2	1.45×10^2	1.89×10^2	2.33×10^2	2.44×10^2	4.71×10^2	4.65×10^1	8.05×10^2	4.01×10^2	9.24×10^2	2.25×10^4	5.06×10^3	6.23×10^3	3.86×10^1	1.63×10^3	3.44×10^2	5.29×10^2
Stem extract 3	1.62×10^2	1.98×10^2	2.00×10^2	2.68×10^2	4.65×10^2	6.34×10^1	7.83×10^2	3.86×10^2	9.47×10^2	1.61×10^4	4.80×10^3	5.52×10^3	3.86×10^1	1.74×10^3	3.26×10^2	5.34×10^2
Trunk extract 1	1.70×10^2	1.68×10^2	2.12×10^2	1.01×10^3	2.30×10^4	2.61×10^3	4.12×10^2	3.89×10^3	1.00×10^4	1.43×10^5	4.06×10^2	3.74×10^2	4.46×10^1	3.49×10^2	5.31×10^1	4.84×10^3
Trunk extract 2	1.46×10^2	1.69×10^2	2.08×10^2	1.13×10^3	2.06×10^4	2.12×10^3	3.62×10^2	3.93×10^3	9.93×10^3	1.46×10^5	4.60×10^2	4.44×10^2	4.18×10^1	2.73×10^2	4.38×10^1	5.06×10^3
Trunk extract 3	1.30×10^2	1.68×10^2	2.21×10^2	9.88×10^2	2.04×10^4	2.54×10^3	3.13×10^2	3.97×10^3	1.07×10^4	1.46×10^5	3.75×10^2	4.11×10^2	3.86×10^1	2.44×10^2	3.95×10^1	5.47×10^3
Fruit oil 1	1.51×10^3	6.25×10^3	5.93×10^3	3.27×10^2	4.61×10^4	5.09×10^3	1.21×10^6	6.57×10^1	5.08×10^1	2.82×10^3	9.03×10^5	1.78×10^6	1.90×10^3	2.82×10^5	9.41×10^4	4.64×10^1
Fruit oil 2	1.50×10^3	6.26×10^3	6.35×10^3	3.16×10^2	5.10×10^4	5.35×10^3	1.02×10^6	6.85×10^1	5.80×10^1	2.42×10^3	1.01×10^6	1.76×10^6	2.16×10^3	2.87×10^5	8.94×10^4	3.89×10^1
Fruit oil 3	1.62×10^3	6.99×10^3	6.46×10^3	3.24×10^2	4.76×10^4	5.54×10^3	1.21×10^6	8.36×10^1	5.79×10^1	2.34×10^3	9.41×10^5	1.64×10^6	2.07×10^3	2.89×10^5	8.93×10^4	3.89×10^1
